# Performance of Serum Biomarkers for the Early Detection of Invasive Aspergillosis in Febrile, Neutropenic Patients: A Multi-State Model

**DOI:** 10.1371/journal.pone.0065776

**Published:** 2013-06-14

**Authors:** Michaël Schwarzinger, Luis Sagaon-Teyssier, Odile Cabaret, Stéphane Bretagne, Catherine Cordonnier, for the PREVERT Investigators, Cécile Pautas, Sébastien Maury, Yosr Hicheri, Françoise Botterel, Francoise Foulet, Anne Vekhoff, Driss Chaoui, Muriel Cornet, Patrice Agnamey, Hassan Farhat, Sylvie Castaigne, Odile Eloy, Felipe Suarez, Agnès Buzyn, Richard Delarue, Svetlana Challier, Nathalie Dhedin, Ahmad Aljijakli, Emmanuelle Delabesse, Annick Datry, Françoise Isnard, Loic Fouillard, Jean-Yves Poirot, Leila Meliani, Lionel Adès, Claire Bouges-Michel, Michèle Deniau, Frédérique Kuhnowski, François Dreyfus, André Paugam, Marie-Thérèse Baixench, Roland Leclercq, Oumady Reman, Chantal Duhamel, Jean-Henri Bourrhis, Sami Chehata, Isabelle Chachati, Vincent Foissaud, Christine Macnab, Hervé Tilly, Stéphane Leprêtre, Christian Gray, Emmanuel Raffoux, Claire Lacroix, Jeremy D Goldhaber-Fiebert, Eran Bendavid, Brandon J Farley

**Affiliations:** Henri Mondor Hospital, Créteil; Henri Mondor Hospital, Créteil; Henri Mondor Hospital, Créteil; Henri Mondor Hospital, Créteil; Henri Mondor Hospital, Créteil; Hôtel Dieu Hospital, Paris; Hôtel Dieu Hospital, Paris; Hôtel Dieu Hospital, Paris; Hôtel Dieu Hospital, Paris; Andre Mignot Hospital, Versailles; Andre Mignot Hospital, Versailles; Andre Mignot Hospital, Versailles; Necker Hospital, Paris; Necker Hospital, Paris; Necker Hospital, Paris; Pitié-Salpétrière Hospital; Pitié-Salpétrière Hospital; Pitié-Salpétrière Hospital; Pitié-Salpétrière Hospital; Saint-Antoine Hospital, Paris; Saint-Antoine Hospital, Paris; Saint-Antoine Hospital, Paris; Saint-Antoine Hospital, Paris; Avicennes Hospital, Bobigny; Avicennes Hospital, Bobigny; Avicennes Hospital, Bobigny; Cochin Hospital, Paris; Cochin Hospital, Paris; Cochin Hospital, Paris; Cochin Hospital, Paris; University Hospital, Caen; University Hospital, Caen; University Hospital, Caen; Gustave Roussy Institute, Villejuif; Gustave Roussy Institute, Villejuif; Gustave Roussy Institute, Villejuif; Hôpital des Armées, Clamart; Hôpital des Armées, Clamart; Centre Henri Becquerel, Rouen; Centre Henri Becquerel, Rouen; Centre Henri Becquerel, Rouen; Saint-Louis Hospital, Paris; Saint-Louis Hospital, Paris; Stanford Health Policy, Stanford University; Stanford Health Policy, Stanford University; University of Provence, Marseille; 1 Equipe ATIP/AVENIR, INSERM, UMR 738, Paris, France; 2 Univ Paris Diderot, Sorbonne Paris Cité, UMR 738, Paris, France; 3 Translational Health Economics Network, Paris, France; 4 Center for Health Policy, Stanford University, Stanford, California, United States of America; 5 INSERM, UMR912 (SESSTIM), Marseille, France; 6 Univ Aix Marseille, UMR_S912, IRD, Marseille, France; 7 ORS PACA, Observatoire Régional de la Santé Provence-Alpes-Côte d’Azur, Marseille, France; 8 Parasitology-Mycology Department, Henri Mondor Teaching Hospital, Assistance Publique-Hôpitaux de Paris (AP-HP), Créteil, France; 9 Univ Paris-Est-Créteil, UMR 956, Créteil, France; 10 Institut Pasteur, Centre National de Référence des Mycoses Invasives et Antifongiques, Paris, France; 11 Hematology Department, Henri Mondor Teaching Hospital, Assistance Publique-Hôpitaux de Paris (AP-HP), Créteil, France; Imperial College, United Kingdom

## Abstract

**Background:**

The performance of serum biomarkers for the early detection of invasive aspergillosis expectedly depends on the timing of test results relative to the empirical administration of antifungal therapy during neutropenia, although a dynamic evaluation framework is lacking.

**Methods:**

We developed a multi-state model describing simultaneously the likelihood of empirical antifungal therapy and the risk of invasive aspergillosis during neutropenia. We evaluated whether the first positive test result with a biomarker is an independent predictor of invasive aspergillosis when both diagnostic information used to treat and risk factors of developing invasive aspergillosis are taken into account over time. We applied the multi-state model to a homogeneous cohort of 185 high-risk patients with acute myeloid leukemia. Patients were prospectively screened for galactomannan antigenemia twice a week for immediate treatment decision; 2,214 serum samples were collected on the same days and blindly assessed for (1->3)- β-D-glucan antigenemia and a quantitative PCR assay targeting a mitochondrial locus.

**Results:**

The usual evaluation framework of biomarker performance was unable to distinguish clinical benefits of β-glucan or PCR assays. The multi-state model evidenced that the risk of invasive aspergillosis is a complex time function of neutropenia duration and risk management. The quantitative PCR assay accelerated the early detection of invasive aspergillosis (*P* = .010), independently of other diagnostic information used to treat, while β-glucan assay did not (*P* = .53).

**Conclusions:**

The performance of serum biomarkers for the early detection of invasive aspergillosis is better apprehended by the evaluation of time-varying predictors in a multi-state model. Our results provide strong rationale for prospective studies testing a preemptive antifungal therapy, guided by clinical, radiological, and bi-weekly blood screening with galactomannan antigenemia and a standardized quantitative PCR assay.

## Introduction

The evaluation of biomarker performance to detect invasive aspergillosis is often limited by the absence of the gold standard for diagnosis and the empirical administration of antifungal therapy for a persistent or recurrent fever. As a case study, patients treated with chemotherapy for acute myeloid leukemia are at high risk of developing invasive aspergillosis that portends poor prognosis [1]. Invasive aspergillosis is rarely proven by direct microbiological detection or autopsy, and often remains a possible or a probable diagnosis depending on a combination of clinical, radiological, and microbiological criteria [2], [3]. Moreover, the risk of invasive aspergillosis evolves over time as it increases with neutropenia duration [4], [5], while it purposely decreases with the empirical administration of antifungal therapy [6]. Accordingly, the performance of biomarkers to detect invasive aspergillosis depends on the timing of test results during neutropenia [7]. The need to evaluate biomarker performance is particularly important in untreated patients because serial blood screening may accelerate the early detection of invasive aspergillosis, independently of other diagnostic information [8]–[11].

*Aspergillus* galactomannan antigen is the biomarker that is most often used in current practice to detect invasive aspergillosis in leukemic patients [12]. Galactomannan antigen detection was added by consensus to the indirect microbiological criteria of a probable invasive fungal disease in 2002 [2]. Serial blood screening with galactomannan antigen has been increasingly used to guide the early administration of antifungal therapy [13], [14] and to monitor treatment [15], [16]. The experience with (1->3)-β-D-glucan (β-glucan) antigenemia or PCR assays is more limited. β-glucan antigenemia was recently incorporated in the definition of a probable invasive fungal disease [3], although β-glucan is not specific of *Aspergillus* spp. [17], [18]. PCR assays targeting *Aspergillus* DNA are even less accepted in routine practice given the lack of common protocols [3].

Meta-analyses found a marked heterogeneity between diagnostic studies for all biomarkers [19]–[23]. Several time-invariant confounders of biomarker performance were consistently identified: distinct features of each biomarker such as the cutoff level used to define a positive test result; the criteria used to define invasive fungal diseases; and the enrollment of patients at different risks of developing invasive aspergillosis. As for time-varying confounders, the definition of a positive test result by two consecutive positive samples drastically reduced heterogeneity between diagnostic studies; however, a more stringent definition implies a lower sensitivity to detect invasive aspergillosis. Overall, the evaluation of biomarker performance according to the timing of test results was underreported in diagnostic studies. Marr et al. found that the sensitivity of galactomannan antigenemia was significantly decreased when anti-mold drugs are administered [24], and the performance of β-glucan antigenemia or PCR assays is thought to be similarly altered [21]–[23].

In the present study, we explored whether β-glucan antigenemia or PCR assays may independently detect invasive aspergillosis when risk management relies on serial blood screening with galactomannan antigenemia. We selected all febrile, neutropenic patients with acute myeloid leukemia from a large clinical trial that included the prospective collection of serum samples twice a week [25]. Because the trial design eventually involved delaying early antifungal therapy in the preemptive arm as compared to the empirical arm [25], we were able to evaluate the performance of β-glucan antigenemia and PCR assays in a homogeneous cohort with extended duration of neutropenia before treatment. We initially evaluated biomarker performance overall, and according to two restrictive approaches with selection of serum samples for evaluation: an early detection approach before treatment, and a confirmatory diagnosis approach in presence of a positive galactomannan antigenemia. Then, we performed a multi-state model describing simultaneously the likelihood of empirical antifungal therapy and the risk of invasive aspergillosis during neutropenia, and we evaluated whether β-glucan antigenemia and PCR assays may accelerate the early detection of invasive aspergillosis when both diagnostic information used to treat and risk factors of developing invasive aspergillosis are controlled for.

## Methods

### Patients

The adult patients evaluated in this study had been enrolled in a prospective, randomized, open-label, non-inferiority trial conducted from April 2003 to February 2006 in 13 French teaching hospitals (ClinicalTrials.gov Identifier: NCT001190463). The primary objective was to compare overall survival following either empirical or preemptive antifungal therapy in patients treated for hematological malignancies. In the empirical therapy arm, persistent or recurrent fever after day 4 of broad spectrum antibacterials led to the administration of antifungal therapy. In the preemptive therapy arm, the initiation of antifungal therapy was guided by clinical and radiological predefined criteria, and bi-weekly blood screening with galactomannan antigenemia. Patients received early antifungal therapy according to the trial protocol, either amphotericin B deoxycholate (1 mg/kg/d) or liposomal amphotericin B (3 mg/kg/d) depending on the level of creatinine clearance and concomitant nephrotoxic drugs. Results of the clinical trial have been reported [25]. The clinical trial and the present study had been approved by the ethics committee of Henri Mondor Teaching Hospital.

Of 293 patients enrolled in the trial, we selected all 185 (63%) febrile, neutropenic patients treated with chemotherapy for acute myeloid leukemia to constitute a homogeneous group at high risk of developing invasive aspergillosis. Exclusions involved: 6 patients without neutropenia; 8 neutropenic patients without any fever; 93 febrile, neutropenic patients treated for hematological malignancies other than acute myeloid leukemia; and 1 patient with acute myeloid leukemia who had no blood sample collected. As compared to excluded patients, the homogeneous cohort selected for the present study had prolonged neutropenia (median duration: 23 days vs. 11 days; P<.001) and a higher risk of developing invasive aspergillosis (11 (6.0%) vs. 1 (0.9%); P = .06), despite a higher rate of early antifungal therapy with intravenous amphotericin B (112 (60.5%) vs. 26 (24.1%); P<.001).

### Antigen and DNA Detection Assays

Blood samples were prospectively collected twice a week during trial enrollment, and processed to serum samples. A portion of serum was immediately screened with ELISA Aspergillus galactomannan antigenemia (Platelia Aspergillus, Biorad, France), and GM index results were available to clinicians within 24 hours for treatment decision according to the trial protocol [25]. The remaining serum was stored at −70°C in two different aliquots.

For the present study, two blinded authors processed all 2,214 serum samples after trial completion. β-glucan was assayed on a specific aliquot using the Fungitell test (Associates of Cape Cod, Inc, Falmouth, MA) according to the manufacturer’s specifications; a positive test result was defined as a level of (1,3)-β-D glucan ≥80 pg/ml. After thawing, DNA extraction was performed from 1 ml serum with the MagNA Pure LC DNA as described previously [26]. A quantitative PCR (qPCR) assay targeting a mitochondrial DNA sequence of Aspergillus was evaluated [27]. This test included the uracil-N-glycosylase use for preventing amplicon contamination, and an internal control based on the amplification of mouse DNA to minimize the risk of false-negative test results [28]–[30].

### Invasive Aspergillosis Case Definition

In the clinical trial, proven and probable invasive aspergilloses were defined by an independent blinded adjudication committee according to EORTC/MSG consensus criteria of 2002 [2]. Invasive aspergillosis was considered as ‘baseline’ for those documented by procedures before or within 24 h after the first dose of early antifungal therapy, and ‘breakthrough’ otherwise.

### Performance of β-glucan Antigenemia and PCR Assays

We initially evaluated biomarker performance as usually done at the patient level. At the core of the evaluation is the definition of a patient tested positive for invasive aspergillosis. We considered that a patient with at least one positive test result is tested positive to maximize the sensitivity of β-glucan, qPCR, or both when used in combination. We evaluated biomarker performance overall with use of all samples collected during study enrollement, and according to two restrictive approaches with selection of serum samples to underline the importance of the timing of test results on biomarker performance: 1) an early detection approach with selection of samples collected after neutropenia or fever onset, and before treatment, invasive fungal disease, or neutropenia recovery; 2) a confirmatory diagnosis approach of a positive galactomannan antigenemia with selection of samples collected on the same sample or the consecutive sample of a GM index ≥0.5.

**Figure 1:**
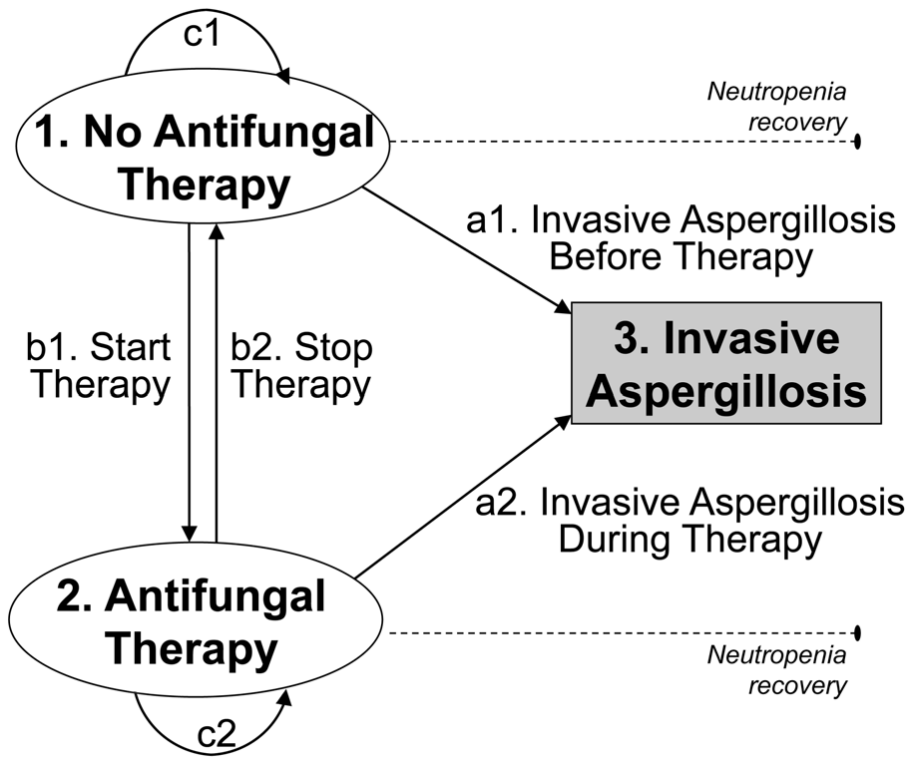
Multi-State Model of Invasive Aspergillosis and Antifungal Therapy in Febrile, Neutropenic Patients with Acute Myeloid Leukemia.

Then, we evaluated biomarker performance as a time-varying predictor at the patient level. At the core of the evaluation is whether the first positive test result with β-glucan or qPCR is an independent predictor of invasive aspergillosis when both diagnostic information used to treat and risk factors of developing invasive aspergillosis are taken into account over time. We developed a multi-state model describing the event history of the patients in continuous time, where events with inherent dependence are defined by the daily transitions between 3 distinct states: (1) ‘no antifungal therapy’ (2) ‘antifungal therapy’ and (3) ‘invasive aspergillosis’ (**Figure 1**). All patients entered the model in the state of ‘no antifungal therapy’ on the first day of neutropenia or fever onset, whichever occurred first, as it may be considered as the starting point of invasive aspergillosis risk in febrile, neutropenic patients. Patients in the initial state free of antifungal therapy could: (a1) develop invasive aspergillosis (i.e., ‘baseline’ invasive aspergillosis); (b1) get treated; or (c1) remain untreated. Once antifungal therapy was started, patients could: (a2) develop invasive aspergillosis despite treatment (i.e., ‘breakthrough’ invasive aspergillosis); (b2) be removed from treatment; or (c2) remain treated. Invasive aspergillosis was the primary outcome of the multi-state model and considered as an absorbing state with no follow-up; otherwise, daily transitions were observed until neutropenia recovery, candidemia or death from causes other than invasive aspergillosis.

A semi-Markov process was used for the multi-state model, where each transition is specified as a separate hazard function and described by an accelerated failure time model. The choice of accelerated failure time models was guided by empirical findings suggesting that each hazard rate depends not only on observed characteristics, but also on the time elapsed in each state: (a1) the risk of invasive aspergillosis increases with neutropenia duration in patients treated with chemotherapy for acute myeloid leukemia [4], [5]; by contrast, (a2) the risk of invasive aspergillosis becomes flat or may even decrease during antifungal therapy as a result of its preventive efficacy [6]; (b1) the likelihood to start empirical antifungal therapy increases with neutropenia duration as clinicians may suspect the risk of invasive aspergillosis and other invasive fungal diseases; (b2) the likelihood to stop empirical antifungal therapy increases with treatment duration as treatment is stopped at neutropenia recovery.

The multi-state model allowed for examination of the independent effect of explanatory variables on each hazard function. We checked whether the first positive galactomannan antigenemia available to clinicians increased the likelihood to start antifungal therapy, and we evaluated whether the first positive test result with β-glucan or qPCR may accelerate the early detection of invasive aspergillosis, i.e., the biomarker was independently associated with an increased risk of invasive aspergillosis before antifungal therapy was started. The evaluation of biomarker performance was controlled for selected confounders on each hazard function. Time-invariant confounders included: age; primary antifungal prophylaxis; and preemptive therapy arm of the trial. Time-varying confounders included: daily assessment of fever above 38°C after day 4 of fever; time to first clinical or radiological pulmonary signs; and timing and length of stay in laminar air-flow rooms or other protective environment rooms with high-efficiency particulate air (HEPA) filters [31]–[33].

### Statistical Analysis

For the evaluation of biomarker performance as usually done, we calculated sensitivity and specificity according to the proportion of patients with true and false positive and negative test results. Ninety-five percent confidence intervals [95% CIs] for sensitivity and specificity were estimated using exact binomial distributions. In addition, we assessed overall biomarker performance by calculating the Youden index (sensitivity+specificity-1) that varies from 0 (no diagnostic accuracy) to 1 (perfect diagnostic accuracy).

For the multi-state model, we estimated simultaneously all four hazard functions under the specifications of each accelerated failure time model and a Weibull parameterization of each baseline hazard [34]–[36]. The family of parametric models as the one used in this analysis not only allows the estimation of the effect of explanatory variables on the hazard rate, but also the estimation of the effect of the time elapsed in each state. The latter effect known as duration dependence is estimated by means of the shape parameter whose value describes how the hazard rate changes over time. A shape parameter equal to 1 indicates a hazard rate that does not vary over time. By contrast, a value above (below) 1 implies a low (high) variability, indicating that patients will transition from one state to another within a relatively short (long) time span. The regression coefficient of an explanatory variable directly measures the proportionate change in the hazard function for a unit change of the explanatory variable, all other things being equal. All analyses were performed using R version 2.11.0 (R Development Core Team, 2010).

## Results

**Table 1** presents the characteristics of the 185 febrile, neutropenic patients with acute myeloid leukemia selected for the present study as well as the risk management of invasive aspergillosis during neutropenia.

**Table 1:** Characteristics of 185 febrile, neutropenic patients treated for acute myeloid leukemia and by invasive aspergillosis.

Characteristic of Patient and Risk Management of InvasiveAspergillosis	Overall,N = 185	InvasiveAspergillosis^a^, N = 11	No Invasive Aspergillosis,N = 174	p-value^b^
**Age, mean (SD), yr**	54 (14)	62 (8)	53 (14)	.030
**Female**	88 (48)	5 (46)	83 (48)	.89
**Remission-induction chemotherapy for new diagnosis or first relapse**	134 (72)	10 (91)	124 (71)	.29
**Duration of neutrophil count <500/mm^3^, median (IQR), days**	23 (16–30)	26 (20–32)	23 (15–30)	.17
**Protective environment room:**				
Laminar air-flow room	63 (34)	1 (9)	62 (36)	
Other protective environment room with HEPA filters	39 (21)	1 (9)	38 (22)	
Non-protective environment room	83 (45)	9 (82)	74 (42)	.013
**Primary antifungal prophylaxis** c	77 (42)	4 (36)	73 (42)	.76
**Antifungal therapy with intravenous amphotericin B** d	112 (61)	4 (36)	108 (62)	.11
**Candidemia**	4 (2)	0	4 (2)	1.00
**Death at end of study** e	8 (4)	2 (18)	6 (3)	.07

Abbreviations: IA: invasive aspergillosis; IQR: interquartile range; HEPA: High-Efficiency Particulate Air.

a Of 11 IA, the independent blinded adjudication committee of the trial defined 2 proven IA and 9 probable IA according to international consensus definitions of 2002.

b By chi-square test or exact Fisher test for binary variables; by Wilcoxon sum-rank test for continuous variables.

c Prophylaxis included oral amphotericin B (n = 54), fluconazole (n = 23), and/or itraconazole (n = 10).

d Of 11 IA, 7 IA were documented by procedures before or within 24 h after the first dose of antifungal therapy, and 4 breakthrough IA occurred after antifungal therapy was started.

e Causes of death included 2 IA, 4 bacterial sepsis, 1 cardiogenic shock, and 1 coma of unknown origin. Follow-up was censored at 14 days after neutropenia recovery or at 60 days of neutropenia.

**Table 2** presents biomarker performance overall, and according to two restrictive approaches with selection of serum samples for evaluation. Overall (median (IQR) of 7 (5–9) samples per patient), all 11 patients with invasive aspergillosis had at least 1 positive test result with β-glucan or qPCR: sensitivity of each biomarker was 82% and 73%, respectively, although specificity was below 50%. In comparison, the selection of serum samples in an early detection approach (median (IQR) of 3 (2–5) samples per patient) was associated with a lower performance of each biomarker before treatment (Youden index below 0.15) as explained by a sharp loss in sensitivity (45% and 36%, respectively). The selection of 59 serum samples in a confirmatory diagnosis approach of a positive galactomannan antigenemia was associated with the best performance of each biomarker (Youden index above 0.45) as explained by an increased specificity above 90%.

**Table 2:** Performance of β-glucan and qPCR, overall and according to two restrictive approaches of invasive aspergillosis diagnosis with selection of serum samples.

Definition of a Patient Tested Positivefor Invasive Aspergillosis		No. patients tested positive/No. patients with IA	Sensitivity, % (95% CI)	No. patients tested negative/No. patients without IA	Specificity, % (95% CI)	Youden index^a^
**Overall performance: all serum samples collected during study enrollment (n = 1,534)**						
	≥1 positive test with ß-glucan	9/11	82 (48–98)	79/174	45 (38–53)	0.27
	≥1 positive test with qPCR	8/11	73 (39–94)	79/174	45 (38–53)	0.18
	≥1 positive test with ß-glucan or qPCR	11/11	100 (72–100)	39/174	22 (16–29)	0.22
**Early detection of IA: selection of serum samples before early antifungal therapy** b **(n = 747)**						
	≥1 positive test with ß-glucan	5/11	45 (17–77)	113/172	66 (58–73)	0.11
	≥1 positive test with qPCR	4/11	36 (11–69)	113/172	66 (58–73)	0.02
	≥1 positive test with ß-glucan or qPCR	7/11	64 (31–89)	76/172	44 (37–52)	0.08
**Confirmatory diagnosis of IA: selection of serum samples in presence of a positive GM test result** c **(n = 59)**						
	≥1 positive test with ß-glucan	6/11	55 (23–83)	159/174	91 (86–95)	0.46
	≥1 positive test with qPCR	6/11	55 (23–83)	162/174	93 (88–96)	0.48
	≥1 positive test with ß-glucan or qPCR	7/11	64 (31–89)	152/174	87 (81–92)	0.51

Abbreviations: IA: Invasive Aspergillosis; GM: Aspergillus galactomannan antigenemia; ß-glucan: (1,3)-b-D glucan antigenemia; qPCR: PCR assay targeting Aspergillus fumigatus mitochondrial DNA.

a Youden index is calculated as follows: Sensitivity+Specificity-1; and varies from 0 (no diagnostic accuracy) to 1 (perfect diagnostic accuracy).

b Selection of serum samples after neutropenia/fever onset, and before antifungal therapy, invasive fungal infection, or neutropenia recovery.

c Selection of serum samples collected on the same sample or the consecutive sample of a GM positive result as defined by a GM index ≥0.5 according to the manufacturer’s specifications.

**Table 3** presents the multi-state model describing the event history of the 185 febrile, neutropenic patients in continuous time. Several observations confirmed the relevance and validity of this approach. The baseline hazard of invasive aspergillosis increased with neutropenia duration before antifungal therapy was started (Weibull shape parameter = 1.89; *P*<.001), and significantly decreased during treatment (Weibull shape parameter = .97; *P*<.001). According to the trial protocol, the empirical administration of antifungal therapy was guided by the diagnostic information available to clinicians: the hazard rate for starting treatment increased with persistent or recurrent fever (acceleration factor = 1.21 [95% CI, 1.16–1.26] for each day of fever after day 4 of broad spectrum antibacterials), the first day of a radiological pulmonary sign (acceleration factor = 1.66 [95% CI, 1.37–2.02]), or the first day of a positive galactomannan antigenemia (acceleration factor = 1.25 [95% CI, 1.11–1.42]); the hazard rate for stopping treatment increased with the first day of fever resolution (acceleration factor = 1.07 [95% CI, 1.01–1.15]). In accordance with trial results, the preemptive therapy arm halved the hazard rate for starting treatment as compared to the empirical therapy arm of the trial (acceleration factor for starting treatment = 0.53 [95% CI, 0.50–0.55]), and treatment duration was consequently decreased before neutropenia recovery (acceleration factor for stopping treatment = 1.19 [95% CI, 1.11–1.28]). In addition, the multi-state model revealed that the risk of invasive aspergillosis before treatment was significantly decreased in laminar air-flow rooms (acceleration factor = 0.34 [95% CI, 0.21–0.55] for each day spent in a laminar air-flow room) as compared to non-protective environment rooms.

**Table 3:** Performance of β-glucan and qPCR to detect invasive aspergillosis in a multi-state model.

	Invasive Aspergillosis Before Therapya1			Start Empirical Antifungal Therapyb1			Invasive Aspergillosis During Therapya2			Stop Empirical Antifungal Therapyb2		
Baseline hazard	Estimate		P-value	Estimate		P-value	Estimate		P-value	Estimate		P-value
**Intercept**	6.85		<.001	2.81		<.001	11.07		<.001	3.49		<.001
**Weibull shape parameter** c	1.89		<.001	2.50		<.001	0.97		<.001	2.30		<.001
**Explanatory variables**	Estimate	CI 95%	P-value	Estimate	CI 95%	P-value	Estimate	CI 95%	P-value	Estimate	CI 95%	P-value
**Age, yr**	1.04	(1.02–1.05)	<.001	0.99	(0.99–0.99)	<.001	1.07	(1.02–1.13)	1.13	1.01	(1.01–1.01)	<.001
**Diagnostic information:**							N/E					
Persistent or recurrent fever	1.17	(0.91–1.50)	.23	1.21	(1.16–1.26)	<.001				0.93	(0.87–0.99)	.023
Clinical pulmonary sign	N/A			1.23	(0.69–2.20)	.49				N/A		
Radiological pulmonary sign	N/A			1.66	(1.37–2.02)	<.001				N/A		
*Aspergillus* GM antigenemia	N/A			1.25	(1.11–1.42)	<.001				N/A		
Preemptive therapy arm of the trial	N/A			0.53	(0.50–0.55)	<.001				1.19	(1.11–1.28)	<.001
β-glucan antigenemia	1.28	(0.57–2.89)	.53	N/A						N/A		
*Aspergillus* qPCR	2.10	(1.20–3.66)	.010	N/A						N/A		
**Preventive interventions other than antifungal therapy:**							N/E					
Laminar air-flow room	0.34	(0.21–0.55)	<.001	0.84	(0.80–0.88)	<.001				0.80	(0.75–0.86)	<.001
Other protective environment room with HEPA filters	0.89	(0.61–1.31)	.56	1.03	(0.98–1.09)	.25				1.28	(1.17–1.39)	<.001
Primary antifungal prophylaxis	1.03	(0.80–1.33)	.80	1.10	(1.05–1.15)	<.001				0.73	(0.68–0.78)	<.001

Abbreviations: GM: Aspergillus galactomannan antigenemia; ß-glucan: (1,3)-b-D glucan antigenemia; qPCR: PCR assay targeting Aspergillus fumigatus mitochondrial DNA; HEPA: High-Efficiency Particulate Air; N/A: not applicable; N/E: not estimable.

a1 Explanatory variables excluded: 1) diagnostic information available to clinicians during the trial because they contribute eventually to the case definition of invasive aspergillosis; 2) the preemptive therapy arm of the trial because its effect is modeled by the decision to start and then stop antifungal therapy during neutropenia.

b1 Explanatory variables excluded β-glucan and qPCR because they were not available to clinicians during the trial.

a2 Explanatory variables included only age because of the limited number of breakthrough invasive aspergillosis (n = 4).

b2 Explanatory variables excluded diagnostic information other than resolution of fever.

c An (exponentiated) Weibull shape parameter above (below) 1 indicates that patients will transition from one state to another within a relatively short (long) time span.

As for the evaluation of biomarker performance as a time-varying predictor in the multi-state model, we found that qPCR assay may accelerate the early detection of invasive aspergillosis, independently of galactomannan antigenemia and other diagnostic information used to start treatment during neutropenia: the first positive test result with qPCR was an independent and significant predictor of invasive aspergillosis before antifungal therapy was started (*P* = .010), while β-glucan was not (*P* = .53). Similar results of biomarker performance were found whether a higher cutoff level (GM index ≥1.5) had been used to define a positive galactomannan antigenemia in serial blood screening (data not shown).

## Discussion

Meta-analyses of biomarker performance to detect invasive aspergillosis found a marked heterogeneity between diagnostic studies [19]–[23]. Among potential sources of heterogeneity, Marr et al. found that the sensitivity of galactomannan antigenemia was significantly decreased by the administration of anti-mold drugs [24], and recommended evaluating biomarker performance in a dynamic approach accounting for the timing of test results [7]. To our knowledge, we present the first multi-state model allowing the evaluation of biomarker performance as a time-varying predictor of invasive aspergillosis before treatment, while multiple sources of heterogeneity may be controlled for. When the multi-state model was applied to a homogeneous cohort of 185 febrile, neutropenic patients screened for galactomannan antigenemia twice a week, we found that PCR assays may accelerate the early detection of invasive aspergillosis before antifungal therapy was started, while β-glucan would not. By contrast, the usual evaluation framework was unable to distinguish clinical benefits of β-glucan or PCR assays.

We confirm that the performance of biomarkers dramatically depends on the timing of test results relative to an evolving risk of invasive aspergillosis. When considering all serum samples collected during study enrollment, the sensitivity of β-glucan (82%) and qPCR (73%) to detect invasive aspergillosis was in the range of pooled estimates (77% [95% CI, 67–84] without difference in the detection of invasive aspergillosis and candidemia [22], and 88% [95% CI, 75–94] [21], respectively), although specificity of both biomarkers (45%) was significantly lower (85% [95% CI, 80–90] for β-glucan [22], and 75% [95% CI, 63–84] for PCR [21]). Such discrepancy may be attributed to the stringent case definition of invasive aspergillosis used in the trial, and false positive test results occurring outside the risk window of invasive aspergillosis, i.e., about half samples were collected before neutropenia/fever onset, during antifungal therapy, or after neutropenia recovery.

In the usual evaluation framework, two approaches allow decreasing false positive rates, while somewhat coping with the timing of test results. As advocated in recent meta-analyses, the definition of a positive test result may be restricted to two consecutive positive samples: specificity improved by about 10% for both β-glucan [23] and PCR assays [21]; however, biomarker sensitivity to detect invasive aspergillosis decreased by about the same extent, and two consecutive samples imply delaying antifungal therapy in patients who should benefit. Alternatively, evaluation may be restricted to serum samples collected in the risk window of invasive aspergillosis. We found that specificity of both biomarkers increased from 45% to 66%, although at the expense of a sharp loss in sensitivity. In addition, when risk management involves blood screening with galactomannan antigenemia twice a week, reserving β-glucan and/or PCR assays for confirming a positive galactomannan antigenemia may be a more cost-effective use of scare resources as the best overall performance was achieved from the drastic selection of these serum samples (4%, 59/1,534).

The usual evaluation framework is misleading on biomarker performance because neither the evolving risk of invasive aspergillosis nor the related kinetics of biomarkers are actually taken into account [7]. The use of serial blood screening was mainly supported by the general finding that the first positive galactomannan antigenenmia precedes clinical and radiological pulmonary signs of invasive aspergillosis [8], [13], [37]. Given the expected correlation of serum biomarkers among each other, serial blood screening would primarily benefit from additional biomarkers that accelerate the early detection of invasive aspergillosis, although the kinetics of biomarkers were barely compared [9]–[11]. Our multi-state model results provide strong rationale for prospective studies to test whether a refined preemptive therapy, guided by clinical, radiological, and bi-weekly blood screening with galactomannan antigenemia and a standardized PCR assay is not inferior to empirical therapy.

Our diagnostic study presents several strengths: a prospective, multicenter design; a homogeneous cohort of 185 febrile, neutropenic patients with acute myeloid leukemia; a central, blinded assessment of serum biomarkers; and the definition of invasive fungal diseases according to EORTC/MSG consensus criteria. As a limitation, the frequency of invasive fungal diseases was relatively low, and we could not model the competing risk of candidemia. However, invasive aspergillosis is the dominant invasive fungal disease in hematological wards, and candidemia is of less concern because of the broad use of primary prophylaxis with anti-yeast activity [38], [39]. Moreover, the evaluation of biomarker performance was controlled for multiple sources of heterogeneity in the risk management of invasive aspergillosis during neutropenia. Besides empirical antifungal therapy, the risk of invasive aspergillosis was significantly decreased in laminar air-flow rooms [31]–[33], while primary prophylaxis may also have been associated with a risk reduction of invasive aspergillosis whether drugs with anti-mold activity had been used [40].

Overall, our study results support the recent call to report the timing of test results in diagnostic studies [21]–[23]. However, the risk of invasive aspergillosis is a complex time function of neutropenia duration and risk management. Accordingly, the comparison of biomarker performance, and more broadly any preventative strategy of invasive fungal diseases, would benefit from the complete reporting of all known time-varying confounders related to the diagnosis and management of invasive fungal diseases.
